# Vitamin D status of psychiatric inpatients in New Zealand’s Waikato region

**DOI:** 10.1186/1471-244X-12-68

**Published:** 2012-06-26

**Authors:** David B Menkes, Kaye Lancaster, Michael Grant, Reginald W Marsh, Peter Dean, Stephen A du Toit

**Affiliations:** 1 Waikato Clinical School, Private Bag 3200, Hamilton, 3240, New Zealand; 2 Waikato District Health Board, Pembroke Street, Hamilton, 3240, New Zealand

## Abstract

**Background:**

Vitamin D deficiency is widespread in New Zealand, confers multiple health risks, and may be particularly common among people with psychiatric illness. We studied vitamin D status in an unselected sample of adult psychiatric inpatients in Hamilton (latitude 37.5 S) during late winter.

**Methods:**

We recruited 102 consenting subjects and measured 25-hydroxy vitamin D3 levels in venous blood using a competitive electrochemiluminescence immunoassay. In addition to descriptive statistics, we used one-sample t-tests to determine the extent to which ethnic and diagnostic subgroups fell below the vitamin D deficiency threshold of 50 nM.

**Results:**

75 subjects (74%) had vitamin D levels <50 nM and thus had at least mild deficiency, while 19 (19%) were severely deficient with levels <25 nM. Rates of deficiency were comparable for men and women; only the former showed a correlation of vitamin D levels with age (r = 0.45, p < 0.01). Maori participants constituted half the sample (n = 51) and were more likely to be deficient than their European counterparts (p = 0.04). Vitamin D also varied by diagnosis, with schizophrenia associated with markedly lower levels than mania and depression (p < 0.001).

**Conclusions:**

Vitamin D deficiency is prevalent in the psychiatric inpatient setting in New Zealand and may be relevant to poor physical health outcomes, notably among Maori and those with schizophrenia. These findings support proposals to provide vitamin D supplementation, particularly during the winter months.

## Background

Vitamin D plays a central role in the regulation of calcium status and is essential for bone health [1]. In the last decade, evidence has accumulated that vitamin D also has other important physiological roles, and is relevant to a variety of clinical conditions, including mental illness. For example, a Finnish cohort of boys receiving supplements during the first year of life showed a decreased risk of developing schizophrenia by age 31 [2]. Vitamin D also appears to influence mood symptoms, based on epidemiological [3] and clinical trial evidence [4].

There is some controversy regarding normal and optimal ranges for vitamin D. Because bone disease is clearly associated with blood levels <25 nM, this threshold is generally accepted as indicating definite deficiency. Levels between 25 and 50 nM are in a grey zone, commonly considered ‘insufficiency’, although some experts insist this still constitutes deficiency [5]. Optimum levels are now considered to be around 75 nM and possibly higher [1,6].

New Zealand’s population appears to have widespread vitamin D deficiency [7-9]; based on its latitude (35-46 degrees S) it is considered to be in a ‘vulnerable’ region [10] with marked seasonal fluctuations in ultraviolet light exposure. In addition, more than 30% of the population has darker skin pigmentation (14.6% Maori; 9.2% Asian; 6.9% Pacific) [11] and among these populations vitamin D deficiency is likely to be even more common, as has been demonstrated in Maori compared to European children [8]. Sunscreen use is prevalent, reducing vitamin D synthesis, and there is little fortification of food [9] compared with North America and Western Europe. These regions also have prevalent deficiency despite repeated calls by authorities to increase the recommended intake of vitamin D [12].

Limited evidence indicates that vitamin D deficiency is common in psychiatric inpatient samples in Germany [13], the UK [14] and Australia [15]; corresponding data from New Zealand have not been reported. However indirect evidence indicates that deficiency is likely to be, if anything, worse than in the general population. Mental disorder is associated with poor diet and limited outdoor exercise, thus tending to limit vitamin D intake and synthesis. Moreover, those with darker skin, particularly Maori, tend both to have higher rates of deficiency and also to be over-represented in psychiatric inpatient populations [16]. Based on the foregoing, we aimed to measure vitamin D levels in psychiatric patients in Hamilton, New Zealand (latitude 37.5^o^S) during the late winter when levels are near their nadir [7,17]. Our secondary aims were to investigate the possible role of ethnicity and diagnosis in rates of vitamin D deficiency in this population.

## Methods

### Subjects and recruitment

The eligible population included 132 adult patients (18–65 years) admitted to the region’s inpatient psychiatric facility between 29 July and 31 August 2010. 102 (78% of those eligible) gave informed consent and had serum samples collected by venepuncture. Ethnicity was determined according to the New Zealand Census procedure 2001, with particular attention paid to evidence of Maori identity by descent. Clinical diagnosis was established at hospital discharge based on DSM-IV criteria. The study was approved by the Northern Y Regional Ethics Committee (NTY/10/EXP/061).

### Biochemistry

Plasma 25-hydroxy vitamin D3 was determined using a competitive electrochemiluminescence immunoassay (E170, Roche diagnostics, Mannheim, Germany). Cross reactivity with 25-hydroxy vitamin D2 and with 24,25-dihydroxy vitamin D is less than 10% and 20% respectively. The coefficient of variation is <8% at each concentration of internal control (35, 50 and 75 nM). Samples were analysed by the Waikato Hospital laboratory, certified by the relevant international accreditation agency (http://www.ianz.govt.nz/). By consensus in New Zealand (http://www.bpac.org.nz/magazine/2011/june/vitamin-d.asp), and in accord with studies mentioned in the Introduction, this laboratory defines levels between 25–50 nM (10–20 ng/ml) and those below 25 nM (10 ng/ml) as indicating mild and severe deficiency, respectively.

### Data analysis

Data were collected using an Excel spreadsheet and analysed using SPSS version 19. One-sample tests were used to determine the extent to which ethnic and diagnostic subgroups fell below the vitamin D deficiency threshold of 50 nM. Parametric correlations and partial correlations were calculated to estimate the effect of age, and Fisher’s Exact Test to identify differences in deficiency rates by category (gender, ethnicity, diagnosis). Cross-tabulation and Cramer’s V test were used to determine the association of psychiatric diagnoses with ethnicity.

## Results

The final sample included 102 subjects, of whom 56 were men and 51 were of Maori ethnicity. Vitamin D levels varied from 10 – 170 nM; mean values by gender, ethnicity, and diagnosis are presented in Table 1. Sub-optimal levels (<75 nM) were detected in 91% of subjects, and at least mild deficiency (<50 nM) in 74%, with comparable proportions of men (70%) and women (76%). Severe deficiency (<25 nM) was seen in 19% overall (16% of men and 22% of women, p = 0.61, Fisher’s Exact Test). There was thus no gender effect regarding overall rates of vitamin D deficiency, but there was a Sex x Age interaction, with men showing significant positive relationship (r = 0.45, p < 0.01) not seen among women (r = 0.11, p < 0.5). Maori men, however, were younger than their non-Maori counterparts by 4 years on average. A partial correlation was calculated to examine the possible role of ethnicity in the observed age effect for men. The resulting comparable correlation (r_parti_ = 0.424, p = 0.002) indicates that the age effect is independent of ethnicity.

**Table 1:** Association of vitamin D levels with gender, ethnicity and diagnosis

**Group**	**n**	**vitamin D (nM)**		**one-sample test**	
		**mean**	**s.d.**	**t**	**p**
Entire sample	102	42.7	21.5	3.45	.001
Male	56	42.6	20.2	2.73	.008
Female	46	42.7	23.2	2.14	.04
Maori	51	37.8	20.3	4.29	<.001
Non-Maori	51	47.5	21.7	0.82	.42
Schizophrenia	38	34.5	15.4	6.23	<.001
Schizoaffective	11	42.8	9.00	2.65	.024
Bipolar Disorder	19	49.4	29.2	0.09	.93
Depression	17	47.5	25.7	0.40	.70
Miscellaneous	17	48.4	20.6	0.32	.75

One sample t-tests were used to estimate the extent to which vitamin D levels in the study population and sub-groups differed from the deficiency threshold of 50 nM.

An ethnic difference in rates of vitamin D deficiency was identified based on 51 patients with verified Maori identity; 14 of these were severely deficient (<25 nM) compared to 5/51 of non-Maori (p = 0.04, Fisher’s Exact Test, see Table 2). As shown in Table 1, mean levels in the Maori group were significantly lower than the non-Maori (mean difference = 9.7 nM, 95% CI = 1.4 to 18.0, t = 2.33, p = 0.022). One-sample statistics showed that Maori were, on average, significantly below the 50 nM deficiency threshold (95%CI = −17.9 to −6.5, t = 4.29, p < 0.001) whereas the same was not true of non-Maori.

**Table 2:** Vitamin D deficiency rates by diagnostic group, ethnicity, and gender

**Group**	**count by Vitamin D level (nM)**				**% less than (nM)**	
	**all**	**>50**	**25–50**	**<25**	**50**	**25**
Entire sample	102	27	56	19	73.5	18.6
Schizophrenia spectrum	49	7	29	13	85.7	26.5
Maori	34	3	20	11	91.2	32.3
male	20	3	11	6	85.0	30.0
female	14	0	9	5	100	35.7
non-Maori	15	4	9	2	73.3	13.3
male	10	4	4	2	60.0	20.0
female	5	0	5	0	100	0.0
Other diagnoses	53	20	27	6	62.3	11.3
Maori	17	4	10	3	76.5	17.6
male	10	3	5	2	70.0	20.0
female	7	1	5	1	85.7	14.3
non-Maori	36	16	17	3	55.6	8.3
male	16	6	9	1	62.5	6.3
female	20	10	8	2	50.0	10.0

Cases were assigned to categories according to vitamin D level; percentages with mild (<50 nM) and severe (<25 nM) deficiency are summarized in the right hand columns.

Vitamin D levels were also found to vary by diagnosis, as shown in Table 1. Among the 19 participants with severe deficiency (<25 nM), 13 had a diagnosis of schizophrenia. This equates to 34% of participants with that diagnosis, compared to 9.4% of other participants (n = 102, p = 0.003, Fisher’s Exact Test). Taken together, disorders in the schizophrenia spectrum (schizophrenia and schizoaffective disorder) were over-represented among those with Vitamin D deficiency (Table 2) and had markedly reduced average levels (n = 49, mean = 36.4 nM, one-sample t = 6.58, p < 0.001) compared to the other diagnostic groups, none of which showed a mean difference from the deficiency threshold of 50 nM (Table 1).

As is apparent in Table 2, Maori appear over-represented in disorders in the schizophrenia spectrum, raising the question of to what extent the effects of ethnicity and diagnosis, described above, may be confounded. We first conducted a cross-tabulation of diagnosis by ethnicity, confirming a highly significant association of Maori ethnicity with schizophrenia and schizoaffective disorder in our inpatient sample (Cramer’s V = 0.415, p < 0.001, n = 102). Comparing ethnicities within the schizophrenia spectrum showed Maori to have a lower mean vitamin D level (34.0 + 14.3 nM, n = 34) than Europeans (41.6 + 14.1 nM, n = 15) but this difference failed to reach statistical significance (t = 1.71, p = 0.093, df = 47). Based on this small sample, it thus appears that Maori ethnicity and schizophrenia are associated with each other, and both contribute to low vitamin D.

Given the finding of excess vitamin D deficiency in schizophrenia, we hypothesized that this might arise from patients with this diagnosis spending relatively less time outdoors. Lacking a specific index of an individual’s sun exposure, we examined time in hospital as a proxy measure. For each participant, we determined days in hospital during the 180 days preceding vitamin D sampling and found a weak relationship in the expected direction between hospitalisation and vitamin D (r = −0.18, n = 102, p = 0.072). Also as expected, patients with schizophrenia and schizoaffective disorder had been on average hospitalised longer (mean = 24.7 + 41.9 days) than other diagnoses (mean = 13.2 + 29.5 days, t = 1.59, p = 0.12) but this difference was not significant and cannot account for the striking association of diagnosis with vitamin D.

## Discussion

Our results indicate a substantial frequency of vitamin D deficiency in a New Zealand inpatient psychiatric population, and accord with evidence of widespread deficiency in the country generally [7-9], and in psychiatric samples overseas [13-15]. In the southern province of Canterbury, the population is generally exposed to less ultraviolet light than Waikato – being an average of 7 degrees further from the equator. A careful epidemiological study in Canterbury found an 89% rate of at least mild deficiency (<50 nM) and 35% severe deficiency (<25 nM) during winter (7). These rates are higher than in our clinical sample, although there is no available Waikato norm for comparison. It is thus possible that our results reflect the general Waikato population.

Previous studies performed in nearby Auckland (latitude 36.5^o^S compared to Hamilton 37.5^o^S) [17,18] found vitamin D deficiency to be more frequent in women than men; in our sample there was only a slight, insignificant excess of women with severe deficiency. Our sample of younger and middle-aged adults (18–65) did however show a correlation of vitamin D levels with age in men but not women, as was found in a Swedish study [19]. This may explain why the Auckland studies, which focused on older adults, found significantly higher levels in men.

We found lower mean vitamin D levels among Maori (Table 1), who were also more than twice as likely as Europeans to have severely deficient levels below 25 nM (Table 2). The generally darker Polynesian skin colour may explain this effect, as was suggested for the comparable difference found in New Zealand children [8]. Low vitamin D is associated with an increased risk of metabolic syndrome, diabetes, and cardiovascular events [20,21]; this may be of particular relevance to Maori whose already elevated cardiometabolic risk appears exacerbated by antipsychotic treatment [22].

Individuals with vitamin D levels below 25 nM may benefit from supplementation, particularly with regard to cardiovascular and bone health [20,21,23], but it is unclear to what extent deficiency and its remediation may affect our patients’ mental disorder. In the case of mood disorder there is suggestive evidence that it may be helpful based on the reported epidemiological association between low vitamin D levels and depressive symptoms [24] and positive results from a trial of supplementation [4].

The observed excess of vitamin D deficiency among patients with schizophrenia and schizoaffective disorder is intriguing. While their slightly greater time in hospital cannot account for this difference, it may be that these patients are generally less likely to spend time outdoors and thus synthesize less of the vitamin. Another possibility, suggested by in vitro studies but yet to verified in patients, is that antipsychotic drug treatment may inhibit vitamin D synthesis [25]. Previous clinical studies have produced mixed results; one found excess vitamin D deficiency in schizophrenia and autism compared to other outpatient diagnoses [19], another reported comparably reduced vitamin D in inpatient schizophrenia and depression [13] and a third showed no difference between patients with psychosis and matched controls [26]. It remains a possibility that vitamin D abnormalities in early life are associated with the pathogenesis of schizophrenia [2,27].

Many countries have high rates of vitamin D deficiency, despite mandatory fortification of foods in, for example, North America, Western Europe, and Australia. Such a policy is yet to be implemented in New Zealand. Supplementation is inexpensive, around $1 per month, and is reported to have few side effects and little toxicity [28] even when large doses are used [29]. At the behest of a government agency (the Accident Compensation Corporation), elderly rest home populations in New Zealand are now routinely supplemented with vitamin D in order to reduce the risk of falls causing fractures and other injuries [23]. There are, as yet, no guidelines on supplementation of psychiatric patients in New Zealand.

## Conclusions

The observed prevalence of vitamin D deficiency in our psychiatric inpatient population supports the idea that supplementation should be more generally available, and perhaps routinely prescribed, given its low cost, lack of adverse effects and multiple potential benefits. It is our experience that ‘natural therapies’ are popular with psychiatric patients; this may facilitate uptake of such treatment, at least during inpatient admission. Supplementation may be of particular benefit to Maori and to patients with schizophrenia; both groups have characteristically poor physical health outcomes and were found in this study to have exceptionally low vitamin D levels.

## Competing interests

The authors declare they have no competing interests.

## Authors’ contributions

KL, PD, SD and DM developed to the original idea and design of the study. KL and MG gained staff support, recruited participants, and collected data; MG also determined ethnicity. SD organized chemical assays, quality control, and reporting. RM conducted the statistical analysis. DM was responsible for overall organization of the study and drafting the manuscript. All authors contributed to editing and approving the final version.

## Authors’ information

David B Menkes, consultant psychiatrist and associate professor

Kaye Lancaster, psychiatric nurse

Michael Grant, trainee psychiatrist of Maori (Te Arawa) ethnicity

Reginald W Marsh, statistician/epidemiologist and honorary associate professor

Peter Dean, consultant forensic psychiatrist

Stephen A du Toit, consultant chemical pathologist

## Pre-publication history

The pre-publication history for this paper can be accessed here:

http://www.biomedcentral.com/1471-244X/12/68/prepub
